# Statistical Methods for Assessing the Explained Variation of a Health Outcome by a Mixture of Exposures

**DOI:** 10.3390/ijerph19052693

**Published:** 2022-02-25

**Authors:** Hua Yun Chen, Hesen Li, Maria Argos, Victoria W. Persky, Mary E. Turyk

**Affiliations:** Division of Epidemiology & Biostatistics, School of Public Health, University of Illinois at Chicago, 1603 West Taylor Street, Chicago, IL 60612, USA; hli226@uic.edu (H.L.); argos@uic.edu (M.A.); vwpersky@uic.edu (V.W.P.); mturyk1@uic.edu (M.E.T.)

**Keywords:** environmental health, estimating equation, linear model, mixture of pollutants, random matrix

## Abstract

Exposures to environmental pollutants are often composed of mixtures of chemicals that can be highly correlated because of similar sources and/or chemical structures. The effect of an individual chemical on a health outcome can be weak and difficult to detect because of the relatively low level of exposures to many environmental pollutants. To tackle the challenging problem of assessing the health risk of exposure to a mixture of environmental pollutants, we propose a statistical approach to assessing the proportion of the variation of an outcome explained by a mixture of pollutants. The proposed approach avoids the difficult task of identifying specific pollutants that are responsible for the effects and may also be used to assess interactions among exposures. Extensive simulation results demonstrate that the proposed approach has very good performance. Application of the proposed approach is illustrated by investigating the main and interaction effects of the chemical pollutants on systolic and diastolic blood pressure in participants from the National Health and Nutrition Examination Survey.

## 1. Introduction

Environmental pollutants are a major source of risk to public health. Evaluating the risks from environmental pollutants is challenging because the pollutants are always mixtures of chemicals and can be highly correlated due to similar exposure pathways and/or chemical structures [1]. In addition, the effect of an individual chemical on a health outcome can be weak and difficult to detect because of the relatively low level of exposures to many environmental pollutants. Recent technological advances allow for measuring a large number of environmental chemicals in biologic and environmental samples. Conventional statistical methods encounter substantial difficulties in analyzing such data where high-dimensional covariates can be highly correlated and the effects of the covariates on the outcome can be weak [2].

One approach to dealing with highly correlated covariates in regression analysis is to apply principal component regression, which uses linear combinations of covariates that explain a large portion of covariate variation as input to the regression model. The principal components method weights each covariate and can be hard to interpret because such combinations are not unique. Factor analysis is traditionally used to improve the interpretation. When data are of high dimension, Zou et al. [3] proposed a sparse principal component analysis that restricts each principal component to sparse non-zero weights by shrinkage approaches such as Lasso [4] or elastic net [5]. One problem with the principal components regression approach is that components accounting for a large portion of the covariate variation do not necessarily explain a substantial proportion of the variation of the outcome. The partial least squares approach [6,7,8] addresses this problem by considering both the covariates and the outcome in forming the components. Chun and Leles [9] proposed a sparse version of the partial least squares that generates linear combinations of sparse covariates for better prediction and interpretation.

When the covariate effects are weak and numerous, it is more attractive to first estimate the variation of the outcome explained by the covariates [10]. Both principal component regression and partial least squares involve choosing the number of components to be included in the regression model. Neither is guaranteed to obtain a stable sparse representation of the components of the regression model even if the linear regression model holds. This prevents us from obtaining an unbiased estimation of the total variation explained by the measured covariates in general. When the sparsity does hold in the linear regression model, Cai and Guo [11], Verzelen and Gassiat [12], and Cai and Guo [13] proposed optimal estimators of the variation explained by the measured covariates. In practice, we may not know if the sparsity assumption holds. In this case, a more attractive estimator of the variation explained by the covariates is to use the normal random-effects model [14]. The method of Yang et al. [14], termed genetic complex trait analysis (GCTA), was proposed for estimating the narrow-sense heritability in the genome-wide association study of single-nucleotide polymorphism (SNPs) effects on a complex trait. The estimator is consistent when the SNPs are independent and the number of SNPs is in the comparable order of the sample size [14]. Dicker [15] studied the asymptotic distribution of a similar estimator under the normality assumption for the independent covariates. Janson et al. [16] proposed an alternative approach termed EigenPrism to construct confidence intervals for the total variation explained by the covariates also under the normality assumption for the independent covariates and the residual errors.

The methods for inference in Yang et al. [14], Dicker [15], and Janson et al. [16] all rely on the normality assumption on data generation. Chen [17] proposed an estimating equation approach that relies neither on the normality assumption of the covariates nor the residual errors. This estimator was shown to be consistent and asymptotically normally distributed under some reasonable conditions. One key assumption is the independence of the covariates in the model. Yet, in the study of environmental pollutants, it is often the case that the pollutants are correlated. To address this problem, we propose to use a special weighting matrix along with strategies for estimating the correlation matrix with possible supplemental data. The proposed approach enables us to estimate the explained variation by the environmental pollutants and to examine interaction effects of those pollutants.

We apply this approach to the analysis of the association of chemical pollutants, in particular persistent organic pollutants (POPs), such as polycholorinated biphenyls (PCBs), on systolic and diastolic blood pressure in a cross-sectional dataset from the National Health and Nutrition Examination Survey (NHANES), 1999–2004. The selection of this health outcome was motivated by previous investigations that demonstrated associations of POPs with prevalent or incident hypertension [18,19,20] and increases in systolic and diastolic blood pressure [21,22] in populations with general exposures, such as NHANES, and those living near highly contaminated areas.

## 2. Material and Methods

### 2.1. Estimation of the Total Effects of Exposures

Denote the health outcome by *Y* and the exposures to the mixtures of pollutants by X, composed of *p* individual chemicals: $X_{1},\cdots ,X_{p}$, i.e., $X={\left(X_{1},\cdots ,X_{p}\right)}^{t}$. Let the mean prediction of the health outcome Y  given the exposures X be $E\left(Y|X_{1},\;\cdots ,X_{p}\right)$. The proportion of the variation of the health outcome explained by the collection of the environmental exposures is defined as

(1) $$r^{2}=\frac{var\left\{E\left(Y|X_{1},\;\cdots ,X_{p}\right)\right\}}{var\left(Y\right)}.$$

If there is no association between the health outcome and the environmental exposures, the mean prediction is a constant, and thus the proportion of the explained variation is zero. When the dimension of the environmental exposures is low, it may be feasible to first estimate the regression model and then assess the proportion of the variation explained. When the dimension of the exposures is high, the approach of estimating the regression model first becomes difficult to impossible to carry out. We propose an alternative approach that directly estimates the proportion of the explained variation without the need to estimate the regression model first. Assume the linear model linking the health outcome and the pollutants as

(2) $$Y=\beta_{0}+\beta_{1}X_{1}+\cdots +\beta_{p}X_{p}+\epsilon ,$$

where $E\left(\epsilon \right)=0,\;var\left(\epsilon \right)=\sigma^{2}$. Let $\beta ={\left(\beta_{1},\cdots ,\beta_{p}\right)}^{t}$ and ∑=var(X). The proportion of explained variation under the linear model (2) becomes

$$r^{2}=\frac{\beta^{t}\sum \beta }{\beta^{t}\sum \beta +\sigma^{2}}.$$

If the covariance matrix ∑ is known, a decorrelation transformation can be applied to transform $X_{1},\cdots ,\;X_{p}\;$ into uncorrelated variables $Z_{1},\cdots ,Z_{p}$ through $\left(Z_{1},\cdots ,Z_{p}\right)=\left(X_{1},\cdots ,X_{p}\right)\sum^{-1/2}.\;$ After the decorrelation transformation, the linear model becomes

(3) $$Y=\beta_{0}+\alpha_{1}Z_{1}+\cdots +\alpha_{p}Z_{p}+\epsilon ,$$

where $\alpha ={\left(\alpha_{1},\cdots ,\alpha_{p}\right)}^{t}$ and $var\left(Z_{1},\cdots ,\;Z_{p}\right)=I_{p}$, the identity matrix. Furthermore, $\alpha^{t}\alpha =\beta^{t}\sum \beta$. The proportion of outcome variation explained by covariates $\left(Z_{1},\cdots ,Z_{p}\right)$ under model (2) can be rewritten as

$$r^{2}=\frac{\alpha^{t}\alpha }{\alpha^{t}\alpha +\sigma^{2}}$$

Under model (3), let the observed outcomes be $Y_{i},\;i=1,\cdots ,n,\;$ and the observed exposures be $\left(Z_{i1},\cdots ,Z_{ip}\right),\;i=1,\cdots ,n.\;$ The proportion of the explained variation $r^{2}$ can be directly estimated by

(4) $${\hat{r}}^{2}=\frac{tr\left[W\left\{\tilde{Y}{\tilde{Y}}^{t}-\left(I_{n}-\frac{11^{t}}{n}\right)\right\}\right]}{tr\left[W\left\{M-\left(I_{n}-\frac{11^{t}}{n}\right)\right\}\right]}$$

where $\tilde{Y}=\left(Y_{1}-\bar{Y,}\;\cdots ,Y_{n}-\bar{Y}\right)/\sigma_{Y}$, $\bar{Y}=\frac{1}{n}\overset{n}{\underset{i=1}{\sum }}Y_{i}$, $\sigma_{Y}^{2}=\frac{1}{n-1}\overset{n}{\underset{i=1}{\sum }}{\left(Y_{i}-\bar{Y}\right)}^{2}$, $1={\left(1,\cdots ,1\right)}^{t}$, and $M={\left(M_{ik}\right)}_{n\times n\;}$ with

$$M_{ik}=\frac{1}{p}\overset{p}{\underset{j=1}{\sum }}\left(Z_{ij}-{\bar{Z}}_{+j}\right)\left(Z_{kj}-{\bar{Z}}_{+j}\right)$$

and ${\bar{Z}}_{+j}=\frac{1}{n}\overset{n}{\underset{i=1}{\sum }}Z_{ij}$, $\mathrm{and}\;W={\left(I+\lambda M\right)}^{-1}\left(M-I\right){\left(I+\lambda M\right)}^{-1}$ for fixed λ≥0. One major advantage of using (4) to estimate the proportion of the explained variation is that it allows for high-dimensional exposures in model (3). The dimension of the exposures *p* can be as large as or even greater than the sample size *n*, as long as the increase in dimension is approximately a linear function of the sample size. Chen [17] showed that the estimator for $r^{2}$ behaves well in the high-dimensional setting.

### 2.2. Decorrelation of the Exposures with Possibly Supplementary Exposure Data

The estimation of the proportion of the explained variation by (4) is based on model (3), which requires the exposures be de-correlated before being used. In practice, the covariance matrix may not be known a priori. One idea is to use the estimated covariance matrix for the decorrelation. However, there are potentially two problems in implementing this idea when the exposures are of high dimension. First, the covariance matrix may not be directly estimable when the dimension is high. For example, the empirical estimate of the covariance matrix does not yield a good estimate of the covariance matrix if *p* is close to or larger than *n*. Second, even if the covariance matrix can be estimated in some ways, the resulting estimator of the proportion of the explained variation may not behave well. We propose the following approaches to address these issues.

When n>p, the covariance matrix can be directly estimated by the empirical estimator as $\hat{\sum }=\left({\hat{\sum }}_{jk}\right)$, where

$${\;\hat{\sum }}_{jk}=\frac{1}{n-1}\overset{n}{\underset{i=1}{\sum }}\left(X_{ij}-{\bar{X}}_{+j}\right)\left(X_{ik}-{\bar{X}}_{+k}\right)$$

and ${\bar{X}}_{+j}=\frac{1}{n}\overset{n}{\underset{i=1}{\sum }}X_{ij},\;j=1,\cdots ,p$. When n≤p, to continue the use of the empirical estimator for the decorrelation, it is required to have additional supplementary covariate data. Suppose a separate sample of covariates only is available to use. Denote the supplementary data by $X_{i1},\cdots ,X_{ip},\;i=n+1,\cdots ,n+N$, where n+N>p. The empirical estimator of the variance matrix $\hat{\sum }=\left({\hat{\sum }}_{jk}\right)$, where

$${\;\hat{\sum }}_{jk}=\frac{1}{n+N-1}\overset{n+N}{\underset{i=1}{\sum }}\left(X_{ij}-{\bar{X}}_{+j}\right)\left(X_{ik}-{\bar{X}}_{+k}\right)$$

and ${\bar{X}}_{+j}=\frac{1}{n+N}\overset{n+N}{\underset{i=1}{\sum }}X_{ij},\;j=1,\cdots ,p$. Note also that if supplementary covariate data are available, the supplementary data can also be used for the covariance matrix estimation when n>p. With the covariance matrix estimated, decorrelation can be carried out by the following approach,

$$\left(Z_{i1},\cdots ,Z_{ip}\right)=\left(X_{i1},\cdots ,X_{ip}\right){\;\hat{\sum }}^{-1/2}$$

i=1,⋯,n. The estimation approach in (4) can then be carried out using the decorrelated covariate data Z in *M* and *W*, denoted, respectively, by $\hat{M}$ and $\hat{W}$. The estimator of the proportion with estimated weight matrix is

(5) $${\hat{r}}_{e}{}^{2}=\frac{tr\left[\hat{W}\left\{\tilde{Y}{\tilde{Y}}^{t}-\left(I_{n}-\frac{11^{t}}{n}\right)\right\}\right]}{tr\left[\hat{W}\left\{\hat{M}-\left(I_{n}-\frac{11^{t}}{n}\right)\right\}\right]}.$$

It has been shown that the empirical estimate of the covariance when n>p and the empirical estimate when n≤p with supplementary exposure data can yield well-behaved estimator of $r^{2}$ by the method in (4) and (5), respectively. More details can be found in Chen (2021). Note, however, current theory does not support the use of other types of covariance estimators in place of the empirical estimator, including the frequently used sparse matrix approaches in high-dimensional settings.

### 2.3. Confounder Adjustment and the Variation Explained by a Subset of Exposures

In estimating the proportion of the variation of a health outcome explained by a set of exposures, it is often the case that some confounders need to be adjusted. Another relevant question is estimating the proportion of the variation of a health outcome explained by a subset of the exposures. In this case, we need to account for the fact that the subset of exposures may be correlated with the rest of the exposures and that the rest of the exposures may also explain a proportion of the variation of the health outcome. These two problems are similar from a statistical point of view. We treat them in a single framework, where we label the confounders and the exposures together by covariates.

Let the set of covariates be divided into two sets, denoted by $X_{A}={\left(X_{j},\;j\in A\right)}^{t}$ and $X_{B}={\left(X_{j},\;j\in B\right)}^{t}$, where A and B are two disjoint index sets. For example, $X_{A}$ can be the collection of confounders and $X_{B}$ be the collection of exposures. Suppose that we are interested in estimating the proportion of the additional variation of the health outcome explained by the set of covariates $X_{B}$ after the subset of covariates $X_{A}$ are already included in the model. The proportional of additional variation explained is

$$r_{B|A}^{2}=\frac{E\left[var\left\{E\left(YX_{A},\;X_{B}\right)X_{B}\right\}\right]}{var\left(Y\right)}.$$

Under the linear model in (6),

(6) $$r_{B|A}^{2}=\frac{\beta_{B}^{t}\left(\sum_{BB}-\sum_{BA}\sum_{AA}^{-1}\sum_{AB}\right)\beta_{B}}{var\left(Y\right)},$$

where the covariance matrix for $\left(X_{A},\;X_{B}\right)$ is

$$\sum ={\left(\;\begin{array}{cc} \sum_{AA} & \sum_{AB}\\ \sum_{BA} & \sum_{BB} \end{array}\right)}_{p\times p\;}.$$

The proportions of the variation of Y explained by $\left(X_{A},\;X_{B}\right)$ and $X_{A}$ alone are, respectively

$$r_{AB}^{2}=\frac{\left(\beta_{A}^{t},\;\beta_{B}^{t}\right)\sum {\left(\beta_{A}^{t},\beta_{B}^{t}\right)}^{t}}{var\left(Y\right)}\;\mathrm{and}\;r_{A}^{2}=\frac{{\left(\beta_{A}+\sum_{AA}^{-1}\sum_{AB}\beta_{B}\right)}^{t}\sum_{AA}\left(\beta_{A}+\sum_{AA}^{-1}\sum_{AB}\beta_{B}\right)}{var\left(Y\right)}.$$

They are related through $\;r_{B|A}^{2}=r_{AB}^{2}-r_{A}^{2}$. Estimation of $r_{B|A}^{2}$ can therefore be obtained by

$${\hat{r}}_{B|A}^{2}={\hat{r}}_{AB}^{2}-{\hat{r}}_{A}^{2},$$

where ${\hat{r}}_{AB}^{2}$ and ${\hat{r}}_{A}^{2}$ are, respectively, the proportions of variation estimated by data on $\left(Y,X_{A},X_{B}\right)$ and on $\left(Y,X_{A}\right)$. The estimation approach can be either the direct estimating equation approach assuming covariate independence, or the supplementary covariate approach allowing correlated covariates.

### 2.4. Estimation of the Total Interaction Effects

One important practical question in environmental health research is whether interactions among pollutants exist. To answer this question, linear model (1) can be expanded to include interactions as follows,

(7) $$Y=\beta_{0}+\beta_{1}X_{1}+\cdots +\beta_{p}X_{p}+\sum_{j=1}^{p-1}\sum_{k=j+1}^{p}\gamma_{jk}X_{j}X_{k}+\epsilon ,$$

For estimating the total variation explained by both the main and interaction effects, the estimator ${\hat{r}}^{2}$ or ${\hat{r}}_{e}^{2}$ may be used depending on whether the covariance matrix needs to be estimated. For estimating the proportion of variation explained by the interaction effects only, the problem can be cast into the covariate adjustment formulation discussed in the previous section. Let $X_{A}=\left(X_{1},\cdots ,X_{p}\right)$ and $X_{B}=\left(X_{1}X_{2},\cdots ,\;X_{p-1}X_{p}\right)$. The proportion of variation explained by the interaction terms given the inclusion of the main effect terms is $r_{B|A}^{2}$. The parameter may be estimated using the method described in the last section.

### 2.5. Inference on the Explained Variation

One important question to ask in practice is whether the explained variation by a mixture of exposures is non-zero. To answer this question, we propose to use the permutation test. Specifically, we first permute the outcomes among the subjects and recalculate the estimated value for $r^{2}$. By comparing the $r^{2}$ estimator based on the permuted data with that from the original data, and repeating the process for many times, we can estimate the *p*-value for testing the hypothesis of no explained variation. Specifically, we test the hypothesis,

$$H_{0}:r^{2}=0\;vs.\;\;H_{A}:r^{2}>0.$$

The following permutation algorithm is used.

1. Compute $r^{2}$ estimator using one of the proposed methods based on data $\left(Y_{1},X_{1}\right),\cdots ,\left(Y_{n},\;X_{n}\right)$. Denote the estimate by ${\hat{r}}_{o}^{2}$.
2. Permute the outcome $Y_{1},\cdots ,Y_{n}$, to $Y_{\tau \left(1\right)},\cdots ,Y_{\tau \left(n\right)}$, where τ is a randomly selected permutation of indices 1,⋯,n. Compute $r^{2}$ estimator using the same method based on permuted data $\left(Y_{\tau \left(1\right)},X_{1}\right),\cdots ,\left(Y_{\tau \left(n\right)},\;X_{n}\right)$. Denote the estimate by ${\hat{r}}_{\tau }^{2}$.
3. Repeat step 2 for N times. The estimated *p*-value is the frequency of ${\hat{r}}_{\tau }^{2}>{\hat{r}}_{o}^{2}$, i.e.,

$$\hat{p}-value=\frac{\#\left\{\tau |{\hat{r}}_{\tau }^{2}>{\hat{r}}_{o}^{2}\right\}}{N}.$$

To take into consideration the accuracy of the Monte Carlo simulation approach in computing the *p*-values, we obtain an upper bound of the true *p*-value by

$$\hat{p}-value\;bound=\hat{p}-value+0.427/\sqrt{N}$$

The second term is obtained from the upper bound of the 95% confidence interval for *p*-value equals 0.05. The *p*-value bound is more appropriate to use when the estimated *p*-value is small in comparison to the accuracy that can be achieved by the Monte Carlo simulation approach.

Another question often asked in practice is whether the explained variation by a mixture of exposures is non-zero after adjusting for confounders. This also includes the additional explained variation of a group of exposures after other groups of exposures are already explained. One such example is the additional variation explained by interactions. The hypothesis to be tested can in general be stated as

$$H_{0}:r_{B|A\;}^{2}=0\;vs.\;H_{A}:r_{B|A}^{2}>0.$$

The following permutation algorithm can be used after the decorrelation transformation:

1. Compute $r_{B|A}^{2}$ estimator using one of the proposed methods based on data $\left(y_{1},X_{A1},\;X_{B1}\right),\cdots ,\left(y_{n},\;X_{An},X_{Bn}\right)$. Denote the estimate by ${\hat{r}}_{B|Ao}^{2}$.
2. Permute the covariates $X_{B1},\cdots ,X_{Bn}$, to $X_{B\tau \left(1\right)},\cdots ,X_{B\tau \left(n\right)}$, where τ is a random selected permutation of indices 1,⋯,n. Compute $r_{B|A}^{2}$ estimator by the same method based on the permuted data $(Y_{1},X_{A1},X_{B\tau \left(1\right)}),\cdots ,\left(Y_{n},X_{An},X_{B\tau \left(n\right)}\right)$. Denote the estimate by ${\hat{r}}_{B|A\tau }^{2}$.
3. Repeat step 2 for N times. The estimated *p*-value is the frequency of ${\hat{r}}_{B|A\tau }^{2}>{\hat{r}}_{B|Ao}^{2}$, i.e.,

$$\hat{p}-value=\frac{\#\left\{\tau |{\hat{r}}_{B|A\tau }^{2}>{\hat{r}}_{B|Ao\;}^{2}\right\}}{N}.$$

Similarly, we can compute an upper bound of the true *p*-value by

$$\hat{p}-value\;bound=\hat{p}-value+0.427/\sqrt{N},$$

to account for the error in using the Monte Carlo approach to computing the *p*-value.

Aside from the permutation tests for no effects, a confidence interval for the explained variation can be constructed based on the asymptotic analysis of the proposed estimators. Such asymptotic results based on the random matrix theory [23] are very complex to obtain. Interested readers may check the results in Chen [17] for more details.

### 2.6. R Package: TEV

We developed an R package called the total explained variation (TEV) for carrying out the computation of the proposed methods. The package includes two main functions for computing the proportion of the explained variations and produces confidence intervals for the explained variation. The first function is named R2ee for computing ${\hat{r}}^{2}$ in (4), the second is named R2eesd for computing ${\hat{r}}_{e}{}^{2}$ in (5) when supplemental covariate data are available. Although ${\hat{r}}_{e}{}^{2}$ appears simply as replacing *M* and *W* in (2), respectively, by $\hat{M}$ and $\hat{W}$, this replacement affects the inference on $r^{2}$. As a result, a different function is used. A third function is the least squares approach for the case n>p, named as R2eels. These functions are modified to perform hypothesis tests based on permutation. The modifications for the unconditional test have PMT attached to their names—for example, R2ee to R2eePMT. The modifications for the performance conditional permutation test have PMTca attached to their names—for example, R2eePMTca. In addition, two existing approaches are also included in the R package for the convenience of comparison. The first is the EigenPrism approach with the function name EigenPrismFull, which is an adaptation of the R function EigenPrism by Jansen et al. [16]. The second is the GCTA approach [14] with the option of bootstrap variance computation [24]. This function is named R2GCTA. The code for TEV is available in the Github site: https://github.com/hychen-uic/TEV (accessed on 30 December 2021).

### 2.7. Simulation Study Design

We simulated exposure data in three different scenarios: independent, mildly correlated, and highly correlated. The distributions of the exposures are either normally or non-normally distributed. The outcome data are simulated following a linear model with random error following normal or non-normal distributions. The simulated data are generated under a combination of parameters, including p,n, independent or correlated exposures with different distributions. The detailed data generation algorithm is given in Appendix A. The first set of simulated data are used to validate the correct size of the permutation tests under the null hypothesis and the adequate power under the alternative hypothesis. The permutation sample size of the Monte Carlo simulations for computing the *p*-values is set to 10,000 except stated otherwise. In the second set of simulations, we compute the confidence intervals and their coverages and lengths for the explained variations when the true values of $r^{2}$ are not zero. Comparison with other existing approaches as well as the variations of the proposed approach is carried out. All the simulation results are based on 1000 replicates except stated otherwise.

### 2.8. Data Analysis Approach

We analyze a National Health and Nutrition Examination Survey (NHANES) dataset to demonstrate the use of the proposed methods for data analysis. NHANES is a weighted sample representative of the U.S. population. Persistent organic pollutants (POPs) were measured in serum from a subgroup of the population in the period 1999–2004. The dataset was downloaded from the NHANES website. The original data have 31,126 records. A total of 75 POPs, including 11 brominated flame retardants, 34 polychlorinated biphenyl (PCB), 13 organochlorine pesticides, and 17 dioxins and furans, are treated as exposure variables in the analysis. When interactions are also considered, an additional 2775 covariate items are included in the analysis, for a total of 2862 covariate items. Analytes with measurements below the limit of detection (LOD) are imputed at the LOD/√2. Since these chemicals are lipophilic, the chemical measurements are all adjusted for serum lipid levels (Phillips et al. 1989). As the POPs were measured only on a subset of the subjects by design, we excluded subjects who were not measured by design. For subjects who were selected for measurement by design, those with missing POPs and confounders were included and missing values were imputed using the R package MICE [25] before the analysis. The final sample used for the illustration has 3261 subjects.

The outcomes we analyze are systolic and diastolic blood pressures. Average systolic and diastolic blood pressures (SBP and DBP, respectively) reported to participants were used for analysis. Blood pressure measures have been adjusted for hypertensive medication use to account for the size of potential treatment effect. For participants currently taking anti-hypertensive medications, we added 10 and 5 mmHg to observed systolic and diastolic blood pressures, respectively [26,27]. Possible confounders, including age, body mass index (kg/m^2^), sex, race/ethnicity (non-Hispanic white, non-Hispanic black, Mexican American, other Hispanic, other race), family poverty income level (<1.3, 1.4–3.4, >3.5), education (less than high school, high school, more than high school), alcohol drinks per year, smoking status (never, former, current), drugs taken last month including hormones modifying drugs (yes, no), adrenal cortical steroids (yes, no), antidiabetic drugs (yes, no), and immunosuppressant drugs (yes, no), are adjusted in the analysis.

The distributional summary of the exposures and the outcomes will be examined first. The proportions of explained variation by all the covariates, including confounders and exposure variables, with/without interactions between the exposures are then estimated and inferred. The proportions of the explained variation by the exposures with/without interactions after adjusting for the confounders are estimated and inferred next. The inference included confidence intervals for the proportion of explained variation and *p*-values for the permutation tests.

## 3. Results

### 3.1. Simulations

**Figure 1:**
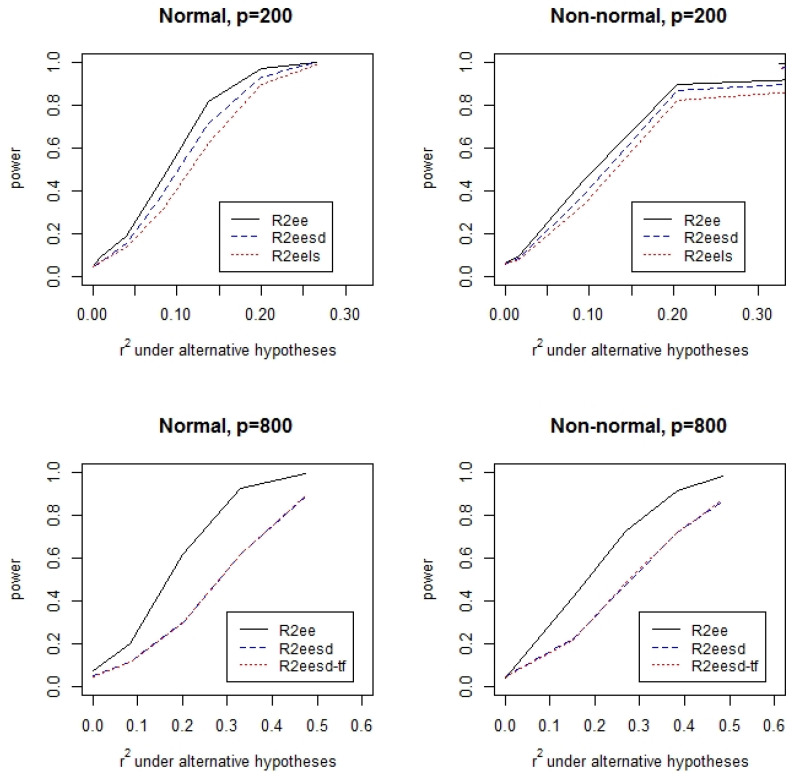
Power of the permutation tests when the covariates are independent.

**Figure 2:**
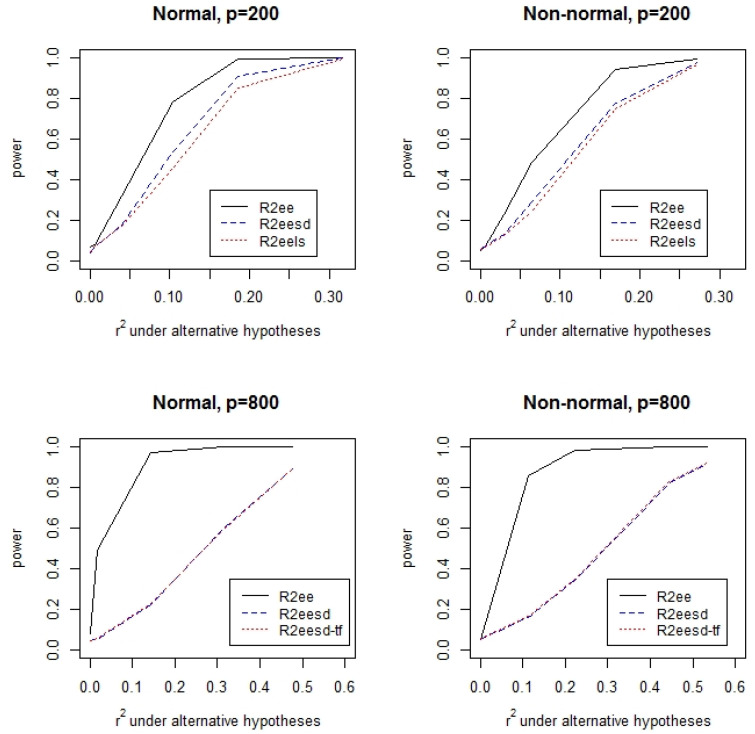
Power of the permutation tests when the covariates are highly correlated.

Table 1 lists the type I errors of the permutation test for the hypothesis $H_{0}:r^{2}=0$ with α=0.05 under varying data generation mechanisms. It can be seen from the table that the type I errors are mostly close to the nominal level. There is a slight inflation of type I error for the test based on the estimating equations assuming independent covariates. For other tests, the type I errors are well under control. Figure 1 and Figure 2 display the power of the permutation tests for the hypothesis $H_{0}:r^{2}=0$ at the significance level α=0.05 with a range of $r^{2}$ values, respectively, for independent covariates and highly correlated covariates. It can be clearly seen that the test that assumes independent covariates has the highest power. The permutation test that uses the supplementary data is a little more powerful than the test that does not use the supplementary data when n>p. The power difference between the test assuming covariate independence and tests without the assumption is large when the ratio of the sample size to the number of covariates is small. For the case with independent covariates (Figure 1), the power of the different tests is similar. In contrast, the power can be substantially different with highly correlated covariates, especially when n<p.

Table 2 lists the estimated proportion of the explained variation by different methods when the proportion is non-zero. From the table, it can be seen that the EigenPrism approach yields reasonably good estimates in terms of bias and variance, and the confidence intervals have good coverage except in the non-normal case with highly correlated covariates. In the latter case, the EigenPrism estimates are subject to large bias and the coverage rates of the confidence intervals are very low. The GCTA approach yields good estimates and confidence intervals when the normality assumption on the random error holds. The GCTA confidence intervals have a low coverage rate when the normality does not hold. Because of the high computation cost in using the bootstrap method for estimating the variance, the GCTA estimator was not computed in the comparison for p>n. Some limited simulation results not shown here suggest the performance is similar to the case of n>p. The confidence intervals of the proposed estimators maintain good coverage for all the simulated cases. The estimating equation approach (R2ee) assuming covariate independence has the shortest length of the confidence intervals. The supplementary data approach (R2eesd) has comparable or wider confidence intervals. This is because the latter approach pays a cost for accounting for the dependence similar to the least squares approach (R2eels) when n>p. It avoids potential bias that may occur in using the R2ee approach for correlated covariates.

Table 3 lists the type I errors of the conditional permutation test for the hypothesis $H_{0}:r_{B|A}^{2}=0$ with α=0.05. This type of test can be used for confounder adjustment, subset exposures, and interactions. Because the simulation is much more time-consuming, the simulation results are obtained with 1000 permutations and 200 replicates. In this set of simulations, $X_{A}$ is set to half of the X variables and $r_{A}^{2}$ is set to either 0 or non-zero. Note in particular that $X_{A}$ is also of high dimension. The estimated type I errors for the proposed test are all under control and close to the nominal level except in some cases of using the R2ee approach.

### 3.2. Data Analysis

**Figure 3:**
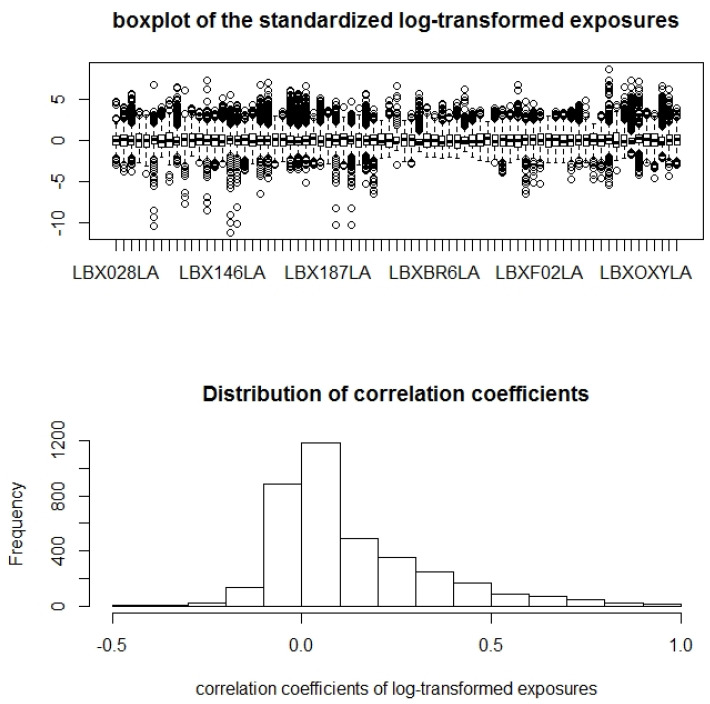
Distributions of exposures and their correlation.

Table 4 lists the summary statistics for the demographic variables and confounders to be adjusted in the analysis. The ranges of the continuous variables appear to be reasonable. The other race categories in the data are small. The percentages of medication use among the subjects are low. Figure 3 shows the box plots of 75 chemical exposures and the distribution of the pairwise correlation coefficients. From the figure, we see that the distributions of the POPs have longer tails than the normal distributions would display. However, the deviations are in general not extreme. The correlation can be high; however, most of the correlation coefficients are approximately 0. Overall, the deviation appears to be not far from independent normal.

Table 5 lists the results of the estimation and inference on the proportions of explained variations. The analysis was performed for the SBP and the DBP, respectively, with/without confounder adjustment and with/without interactions. For the unadjusted analysis without interactions, the proportion of the SBP variation (approximately 35%) explained by the chemical exposures is much higher than that of the DBP (6~7%). Both are significantly different from 0 as the 95% confidence intervals suggest. After the adjustment for the confounders, the proportions of the variation of both the SBP and DBP explained by the POPs is close to 3%, with the DBP slightly less, but significantly different from 0 as the permutation results suggest. Different methods yield very similar results in these cases.

For the model with interactions, the unadjusted estimates from the estimating equation approach assuming independent covariates (R2ee) and the GCTA method are markedly different from the EigenPrism and the least squares approaches. When compared with the estimates without interaction, the R2ee and GCTA approaches do not yield any evidence against no interaction assumptions among the chemical exposures for either the SBP or the DBP. However, both the R2eesd and R2eels approaches yield strong evidence (*p*-value < 0.031) against no interaction assumptions for both SBP and DBP. The least squares type of estimates are considered more reliable because they account for the possible correlations among covariates while the former methods do not. The conditional permutation tests suggest the interactions explain an additional proportion of variation for both the SBP (estimate = 13.2%) and the DBP (estimate = 21.6%).

## 4. Discussion

The proposed methods allow us to infer the proportion of the variation of an outcome explained by a set of exposures collectively with possible adjustment for confounders. One prominent feature of the approaches is that they do not need to first estimate the effects of individual exposures for estimating the overall effects. This feature is useful because it can be hard to estimate the individual effects in high-dimensional exposures. The proposed methods can be applied to situations where either or both the exposures and the confounders are of high dimension where traditional regression approaches do not yield a good estimate of the effects.

There are a number of subtle points which need some attention in applying the proposed methods. Strictly speaking, the proposed methods are applicable for situations with independent covariates before or after a decorrelation transformation. There is a subtle difference between the independence of covariates and the uncorrelated covariates. Theory guarantees for the former but not necessarily the latter. When n≤p and no supplementary covariate data are available, we may impose structures on the covariance matrix so that the covariance matrix can still be reasonably estimated. One such an assumption is the sparsity assumption on the off-diagonal elements for the precision matrix $\sum^{-1\;}$ [28]. However, current theory does not directly support application to cases with an estimated structured covariance matrix. Confounder adjustment in low-dimensional cases can be carried out by regressing on both the outcome and the exposures. In the high-dimensional cases, this adjustment can be impractical. The proposed methods are not subject to the problem.

Data analysis is presented as an illustrative example for applying the proposed methods, rather than an in-depth analysis of the specific dataset. A number of limitations exist with respect to interpreting the results as a substantive analysis of the dataset. First, the missing values are imputed. We performed the analysis only on a single imputed dataset and did not account for the variation in the imputed values. In addition, the imputation model may also be subject to questions and bias may occur for using inappropriate models. Second, the confounder adjustment does not exhaust all possible available information. Additional variables may need to be included, such as use of anti-lipid medications that may impact blood pressure and lipophilic chemical exposures. Furthermore, there may be residual confounding due to additional chemical exposures that may affect blood pressure, such as heavy metals. However, we excluded the heavy metals from the analysis because of a sampling design issue in which some metals and POPs were measured in non-overlapping subsamples. Third, we did not account for the survey sampling design and weights in the analysis.

The proposed approach is currently limited to the continuous outcome under the linear model formulation. For categorical outcomes, the generalized linear model is often used instead, and adaptation of TEV to the more general outcomes is not currently developed. The detailed theory for supporting the use of the proposed methods in this article is not presented in this paper. However, such theory has been developed by the leading author and his collaborators. Interested readers may find it in the reference. One remaining theoretical gap is the lack of a variance formula for the conditional effects estimator for constructing confidence intervals for the proportion of the explained variation after adjustment for covariates. As a result, inference on the total effects relies on the conditional permutation tests only.

We have developed the R code for performing the analysis presented in this paper. We are currently packaging the code into an R package. The package will be posted online for free access. The computations are mostly fast for the proposed methods except for the conditional permutation tests which can be slow when the dimension of the covariates is very high and an accurate *p*-value is to be computed by the simulation.

## 5. Conclusions

The main achievement of the proposed methods is to provide a tool for inferring the overall effects of a large number of exposures on an outcome, with possible adjustment for a large number of confounders, without the need to estimate individual effects of the exposures. Unlike many ad hoc approaches for estimating the overall effects, the proposed approaches have a solid theoretical foundation to guarantee their performance under the condition that covariates can be linearly transformed into independent variables. R code is also available for other researchers to use the proposed methods.

## Figures and Tables

**Table 1:** Estimated type I errors for testing hypothesis *H*_0_: *r*^2^ = 0 by permutation tests with *α* = 0.05.

(n,p)	Tests	N,I	N,M	N,H	C,I	C,M	C,H
(400, 200)	R2ee	0.044	0.053	0.068	0.063	0.059	0.050
	R2eesd	0.046	0.059	0.040	0.056	0.049	0.055
	R2eels	0.046	0.052	0.044	0.061	0.050	0.057
(400, 800)	R2ee	0.075	0.050	0.082	0.041	0.057	0.054
	R2eesd	0.054	0.048	0.048	0.047	0.051	0.051
	R2eesd-tf	0.046	0.046	0.045	0.048	0.057	0.059

N(C), I(M,H): normal ($\mathrm{or}\;\chi_{1}^{2}$) distributed exposures and random error, exposures are independent (or mildly correlated or highly correlated). R2eesd-tf is R2ee with covariates transformed by decorrelation using the estimated covariance with supplementary covariate data.

**Table 2:** Comparison of estimators for the proportion of the explained variation for simulations.

** (n,p) **	**Covariates**	Model $(\mathrm{True}\;r^{2}$)	**Method**	${\hat{r}}^{2}-r^{2}$	Empirical Variance	Averaged 95% CI	95% CI Coverage	95%CI Length
(400, 200)	Independ.	Normal	EigenPrism	0.000	0.0031	(0.263, 0.503)	97.4%	0.240
		(0.401)	GCTA	0.005	0.0030	(0.272, 0.510)	96.5%	0.238
			R2ee	0.000	0.0029	(0.300, 0.501)	94.1%	0.202
			R2eels	0.003	0.0031	(0.285, 0.522)	91.7%	0.237
			R2eesd	0.001	0.0029	(0.298, 0.507)	94.5%	0.209
		$\chi_{1}^{2}$	EigenPrism	−0.004	0.0050	(0.284, 0.517)	89.3%	0.233
		(0.421)	GCTA	−0.003	0.0050	(0.293, 0.528)	89.9%	0.235
			R2ee	−0.003	0.0047	(0.248, 0.589)	97.6%	0.342
			R2eels	−0.001	0.0050	(0.241, 0.608)	94.0%	0.367
			R2eesd	−0.001	0.0048	(0.253, 0.589)	97.1%	0.336
	Correlated	Normal	EigenPrism	−0.002	0.0027	(0.328, 0.547)	95.7%	0.219
		(0.455)	GCTA	−0.017	0.0024	(0.313, 0.547)	97.6%	0.234
			R2ee	−0.020	0.0016	(0.367, 0.506)	89.3%	0.139
			R2eels	−0.002	0.0026	(0.348, 0.560)	92.3%	0.212
			R2eesd	−0.003	0.0026	(0.356, 0.551)	93.8%	0.195
		$\chi_{1}^{2}$	EigenPrism	0.017	0.0052	(0.300, 0.527)	86.3%	0.228
		(0.413)	GCTA	−0.003	0.0049	(0.285, 0.520)	90.5%	0.236
			R2ee	−0.002	0.0035	(0.231, 0.593)	97.8%	0.362
			R2eels	0.020	0.0052	(0.255, 0.617)	89.7%	0.362
			R2eesd	0.019	0.0051	(0.267, 0.597)	93.8%	0.331
(400, 800)	Independ.	Normal	EigenPrism	−0.001	0.0103	(0.160, 0.646)	98.5%	0.485
		(0.403)	R2ee	−0.006	0.0097	(0.207, 0.589)	94.9%	0.382
			R2eesd	0.021	0.0217	(0.146, 0.726)	95.5%	0.580
		$\chi_{1}^{2}$	EigenPrism	0.001	0.0129	(0.183, 0.668)	97.0%	0.485
		(0.423)	R2ee	−0.005	0.0129	(0.182, 0.661)	96.2%	0.479
			R2eesd	0.013	0.0251	(0.135, 0.770)	96.0%	0.635
	Correlated	Normal	EigenPrism	−0.361	0.0036	(0.000, 0.313)	13.4%	0.313
		(0.410)	R2ee	0.034	0.0029	(0.349, 0.538)	82.0%	0.189
			R2eesd	0.020	0.0217	(0.151, 0.730)	94.8%	0.579
		$\chi_{1}^{2}$	EigenPrism	−0.335	0.0034	(0.000, 0.306)	18.8%	0.306
		(0.380)	R2ee	0.048	0.0050	(0.214,0.645)	94.9%	0.431
			R2eesd	0.033	0.0251	(0.117,0.748)	95.8%	0.631

**Table 3:** Estimated type I errors for testing hypothesis $H_{0}:\;r_{B|A}^{2}=0$ by permutation tests in simulations with α=0.05 for (n, p)=(400, 200).

Tests	N,I	N,I	C,I	C,I	N,H	N,H	C,H	C,H
$r_{A}^{2}$	0	0.198	0	0.195	0	0.212	0	0.322
R2ee	0.015	0.065	0.055	0.050	0.105	0.005	0.075	0.005
R2eesd	0.030	0.045	0.065	0.055	0.045	0.015	0.070	0.045
R2eels	0.035	0.025	0.060	0.060	0.055	0.006	0.040	0.040

N(C), I(H): Normal (or $\chi_{1}^{2}$) distributed exposures and random error, exposures are independent (or highly correlated).

**Table 4:** Summary statistics for confounders in the adjustment of the NHANES dataset.

Continuous Variable	Range	Mean	Standard Deviation
Age (in years)	Min = 20, Max = 85	51.82	18.59
BMI	Min = 16.07, Max = 62.99	28.43	5.98
Alcohol drinks/year	Min = 0, Max = 365	48.13	90.36
Categorical variable	Categories	Counts	Frequencies
Gender	Male	1667	0.51
	Female	1595	0.49
Race	Mexican American	733	0.22
	Other Hispanic	132	0.04
	Non-Hispanic White	1722	0.53
	Non-Hispanic Black	573	0.18
	Other race	102	0.03
Education	Less than high school	1060	0.32
	High school diploma	766	0.23
	More than high school	1436	0.44
Poverty/income ratio	Less than 1.3	886	0.27
	Between 1.3 and 3.5	1321	0.40
	More than 3.5	1055	0.32
Smoke status	Never	1637	0.5
	Former	975	0.3
	Current	650	0.2
Taken hormones modifying drugs last month	Yes	584	0.18
	No	2678	0.82
Taken adrenal cortical steroids drugs last month	Yes	74	0.02
	No	3188	0.98
Taken antidiabetic drugs last month	Yes	304	0.09
	No	2958	0.91
Taken immunosuppressant drugs last month	Yes	16	0.005
	No	3246	0.995

**Table 5:** Proportion of blood pressure variations explained by 75 persistent organic pollutants measured in the NHANES dataset.

Outcome	Interaction	Method	Unadjusted	95% CI	Adjusted *	*p*-Value
SBP	No	EigenPrism	0.348	(0.314, 0.379)		
		GCTA	0.348	(0.276, 0.417)		
		R2ee	0.351	(0.319, 0.382)	0.036	0.0044
		R2eels	0.348	(0.313, 0.383)	0.033	0.0044
	Yes	EigenPrism	0.479	(0.398, 0.544)		
		GCTA	0.349	(0.297, 0.403)		
		R2ee	0.349	(0.306, 0.392)	0.000 **	0.090
		R2eels	0.480	(0.413, 0.548)	0.132 **	<0.031
DBP	No	EigenPrism	0.060	(0.012, 0.105)		
		GCTA	0.073	(0.046, 0.105)		
		R2ee	0.073	(0.021, 0.126)	0.034	0.0045
		R2eels	0.061	(0.013, 0.108)	0.023	0.0044
	Yes	EigenPrism	0.275	(0.158, 0.369)		
		GCTA	0.121	(0.072, 0.173)		
		R2ee	0.121	(0.054, 0.189)	0.048 **	<0.031
		R2eels	0.277	(0.179, 0.375)	0.216 **	<0.031

* Adjusted for age, BMI, sex, race/ethnicity, alcohol use, smoking, poverty income level, education, and medication use (see Table 4). ** Permutation tests for no interaction effects adjusted for both confounders and main exposures.

## Data Availability

Data from this work are available in the NIEHS PRIME GitHub: https://github.com/niehs-prime (accessed on 30 December 2021).

## Appendix A

To generate the simulated data, a correlation matrix is first generated as follows. A p×p matrix with independent normal N(a,1) entries and a p×p matrix with independent uniform U[−0.5,0.5] entries are generated. Denote them, respectively, by A and B. Let $D={\left(AB\right)}^{t}AB$. A correlation matrix *C* is extracted from the covariance matrix *D* using the singular value decomposition approach. The level of correlation can be adjusted by changing the values of *a.* In the simulation, independence corresponds to D=I and mild high correlations corresponding, respectively, to a=0 and a=2. Covariates at different levels of correlation are then generated in the following way. Standard normal random numbers are generated first. Power transformation is then applied to generate deviation from the correlated normal, where power transformation maps a random variable u into $u_{1}$ through

(A1) $$u_{1}=sign\left(u\right){\left|u\right|}^{\gamma }$$

where γ>0. In the case of $\chi_{1}^{2}$ covariates, the transformation drops sign(u). The covariates are rescaled to a standard deviation of 1 before being transformed by $C^{1/2}\;$. The covariate effects are set to a fixed constant for half of the variables and 0 for the other half. The constant is chosen along with the error variance so that the proportion of explained variation is close to 0.2, 0.5, 0.8, respectively. Random errors are generated as mean 0 normal random numbers with a possible power transformation to yield deviation from the normal distributed random error. Supplementary covariates with sample sizes equal to the number of covariates are simulated for each case.
